# Outcomes of patients with clinical stage IV esophageal squamous cell carcinoma treated initially with definitive chemoradiotherapy: a single-institution observational study and literature review

**DOI:** 10.1007/s00595-025-03087-x

**Published:** 2025-07-04

**Authors:** Kotaro Sugawara, Koichi Yagi, Shoh Yajima, Yoshiyuki Miwa, Shuichiro Oya, Asami Okamoto, Raito Asaoka, Hideomi Yamashita, Yoshifumi Baba

**Affiliations:** 1 Department of Gastrointestinal Surgery, Graduate School of Medicine, The University of Tokyo, 7-3-1 Hongo, Bunkyo-Ku, Tokyo, 113-8655 Japan; 2 Department of Radiology, Graduate School of Medicine, The University of Tokyo, Tokyo, Japan

**Keywords:** Esophageal squamous cell carcinoma, Clinical stage IV, Definitive chemoradiotherapy, Salvage surgery

## Abstract

**Purpose:**

To investigate the long-term outcomes of patients with cStage IV esophageal squamous cell carcinoma (ESCC) treated with definitive chemoradiotherapy (dCRT) and the impacts of this treatment on inflammatory and nutrition markers.

**Methods:**

The subjects of this study were 84 patients who underwent initial dCRT for cStage IV (cT4 and/or cM1 according to eighth UICC staging system) esophageal squamous cell carcinoma (ESCC). Survival outcomes were investigated according to treatment modalities. Various inflammatory and nutrition markers, such as the C-reactive protein (CRP)-to-albumin ratio (CAR) and the lymphocyte-to-CRP ratio (LCR), were evaluated.

**Results:**

The 3-year overall survival (OS) rate of the 84 patients was 45.8%. Clinical complete response (CR) to dCRT was achieved in 30 patients (dCRT-CR group). Salvage surgery was performed for 35 patients and curative (R0) resection was achieved in 28 patients (surg-R0 group). Patients in the surg-R0 group exhibited comparable 3-year OS (60.7%) to patients in the dCRT-CR group (60.0%). CRP-derived markers (LCR and CAR) were significantly associated with the response to dCRT (both *P* < 0.01) and OS (both *P* < 0.01).

**Conclusions:**

Definitive chemoradiotherapy is appropriate for patients with cStage IV ESCC. Curative salvage surgery provides survival benefits for the tumor entity. Pre-therapeutic CRP-derived markers are useful for predicting the response to dCRT and long-term outcomes.

**Supplementary Information:**

The online version contains supplementary material available at 10.1007/s00595-025-03087-x.

## Introduction

Although multimodal therapies for esophageal squamous cell carcinoma (ESCC) have improved [1], the survival outcomes of patients with initially unresectable (cStage IV, cT4b and/or cM1b) ESCC remain extremely poor [2]. Definitive CRT (dCRT) has been given as initial treatment for patients with cT4b ESCC [3, 4], and investigators have reported 3-year survival rates of 15–33% for patients with unresectable ESCC [5–9]. However, residual or recurrent carcinoma following dCRT is reportedly seen in 40–75% of patients, which is a major issue [2, 10]. For remnant or relapsed EC after dCRT, salvage surgery (SALV) can offer a chance of long-term survival; however, SALV is a highly invasive procedure with considerable morbidity [10, 11]. Prior studies have suggested that SALV outweighs the risks in patients with cT4 ESCC [10, 12–14]; however, it remains unclear whether SALV is beneficial for patients with cT4 ESCC. Recent clinical and observational studies have shown induction chemotherapy and conversion surgery (CS) to yield relatively high conversion surgery rates (42–84%) and to achieve a 3-year survival rate of 46.6% [15–19].

We summarized prior studies on the treatment outcomes of dCRT, SALV, and induction chemotherapy followed by surgery for unresectable ESCC (Table 1). Despite these studies, the optimal treatment strategy for patients with unresectable ESCC remains to be established [17]. Furthermore, pre-therapeutic factors which can help predict the survival outcomes of patients with unresectable ESCC have yet to be fully addressed [13, 20], although several post-therapeutic factors are reportedly associated with survival outcomes in the tumor entity: curative resection [3, 4, 14], response to the induction treatment [20], and lymph-node (LN) involvement [14].

**Table 1:** Summary of studies on locally advanced unresectable esophageal squamous cell carcinoma

Initial therapy	Ref no.	Author	Year	Design	*N*	RT dose	chemotherapy	cCR	CS rate	R0 rate	pCR rate	MST	OS
dCRT	5	Ohtsu	1999	Phase II	54	60	CF	25%				9 m	3-year: 23%
		Nishimura	2002	Phase I/II	28	60	CF	32%				5 m	2-year: 23%
	7	Ishida	2004	Phase II	60	60	CF	15%				10 m	2-year: 32%
		Higuchi	2014	Phase II	42	50.4/61.2	DCF	30/60%				29 m	3-year: 44%
	9	Shinoda	2015	Phase II	68/71	60/60	CF standard/low dose	1/0%				14/13 m	3-year: 26/26%
	8	Miyazaki	2015	Phase I/II	37	60	DCF	48%				53 m	3-year: 44%
	6	Jingu	2016	Observational	128	50–70	DCF, CF, 5-FU + NDP						2-year: 32.8%
		Satake	2016	Phase I/II	33	60	DCF	39%				26 m	3-year: 79%
	10	Sugawara	2019	Observational	73	50–65.4	CF, TS-1 + NDP	29%		71%	39%	21.3 m	3-year: 40.8%
	12	Ohkura	2019	Observational	33	50.4–71.4	CF, DCF			42%	9%		5-year: 63.6% (R0)
	13	Booka	2020	Observational	18	60	CF, 5-FU + NDP			78%	28%	90.1 m	5-year: 51.6%
	14	Okamura	2020	Observational	35	50–70	CF			54%		8.7 m	5-year: 5.7%
		B. Chen	2021	Observational	136	50–64	CF, PC	21%				12.2 m	5-year: 20.2%
Induction chemotherapy		Ikeda	2001	Phase II	37	30	CF		35%	32%	3%	10 m	5-year: 23%
		Fujita	2005	Observational	26	36	CF		58%	42%	4%	22 m	3-year: 23%
	19	Shimoji	2013	Observational	87	40–66	5-FU + NDP ± ADM		70%	61%	14%	10/26 m	5-year: 35%
	15	Yokota	2016	Phase II	48	50.4	DCF (+ RT)		42%	40%	20%		1-year: 68%
	16	Sugimura	2021	Phase II	99	50.4	CF, DCF (+ RT)		84/84%	78/76%	40/17%		
	17	Yamazaki	2023	Phase II	99	50.4	DCF + RT/DCF		84/84%	78/76%	40/17%		2-year: 55.1/34.7%
	18	W. Chan	2023	Observational	47	50	DCF (+ RT)		51%	63%			2-year: 53.3%

*dCRT* definitive chemoradiotherapy, *CF* cisplatin + 5-FU, *DCF* docetaxel + cisplatin + 5-FU, *NDP* nedaplatin, *PC* paclitaxel + cisplatin, *CR* complete response, *CS* conversion surgery, *MST* mean survival time, *OS* overall survival

Growing evidence suggests that pre-therapeutic peripheral blood-derived systemic inflammatory response (SIR) and nutritional markers influence the survival of EC patients [21, 22]. Recently, the CRP-based SIR markers, C-reactive protein (CRP)-to-albumin ratio (CAR) and the lymphocyte C‐reactive protein ratio (LCR), were proposed as new prognostic markers in patients with various malignancies [23] including EC [24, 25]. We conducted this study to investigate the long-term outcomes of patients with cStage IV ESCC, who were treated initially with dCRT, and to evaluate the survival impacts of various SIR and nutritional markers.

## Patients and methods

### Patients

Between 2011 and 2021, 84 patients with clinical stage IV (cT4 and/or M1 according to the eighth edition of the Union for International Cancer Control (UICC) staging system [26]) pathologically confirmed ESCC were commenced on dCRT at the University of Tokyo Hospital. M1b disease included pretracheal (106pre) LN metastasis, paraaorta LN metastasis and intramural gastric metastasis. At the time of the final follow-up (October, 2024), the median follow-up period was 93.2 months for the survivors. This retrospective study was approved by the local ethics committee of the faculty of medicine at the University of Tokyo (ID: 3962).

### Clinical staging

The clinical stage was established in accordance with the findings of esophagogastroduodenoscopy, computed tomography (CT), and/or positron emission tomography. Clinical stage was assessed by members of a multidisciplinary team according to the eighth edition of the UICC staging system [26]. Involvement of adjacent organs was identified by endoscopy or CT, as described previously [11, 27]. Briefly, tracheobronchial invasion was diagnosed if protrusion of the tumor into the lumen of the trachea or bronchus was visualized on CT. When the fat plane in the triangular space between the esophagus, aorta, and spine was diminished, or the degree of direct contact between the tumor and aorta exceeded 90 degrees on CT, aortic invasion was diagnosed. Clinical LN metastasis was defined if LNs of 8 mm or larger were identified on CT images.

### Definitive chemoradiotherapy

The choice of treatment strategy was discussed at a biweekly multidisciplinary cancer board meeting. Definitive CRT was performed as described previously [11]. All patients underwent concurrent platinum-based chemotherapy and radiotherapy with a total radiation dose of 50.4–65.4 Gy delivered in 1.8–2 Gy per fraction over 6–7 weeks. Two-to-four courses of chemotherapy combining 5-fluorouracil or oral S-1 with cisplatin or nedaplatin were administered, as described previously [28]. After completing dCRT, all patients were restaged by endoscopy, CT, and/or PET-CT to evaluate the clinical response based on the criteria of the Japanese Esophageal Society [29, 30]. Complete response (CR) was defined as follows: the disappearance of endoscopic findings suggesting the presence of a tumor, confirmation of negative endoscopic biopsy findings, and the disappearance of all target lesions. Partial response (PR) and progressive disease (PD) were defined as at least a 30% decrease and at least a 20% increase in the sum of the greatest dimensions of target lesions, respectively. Stable disease (SD) was diagnosed in non-CR patients with neither PR nor PD.

### Treatment strategy and surgical procedure

**Fig. 1:**
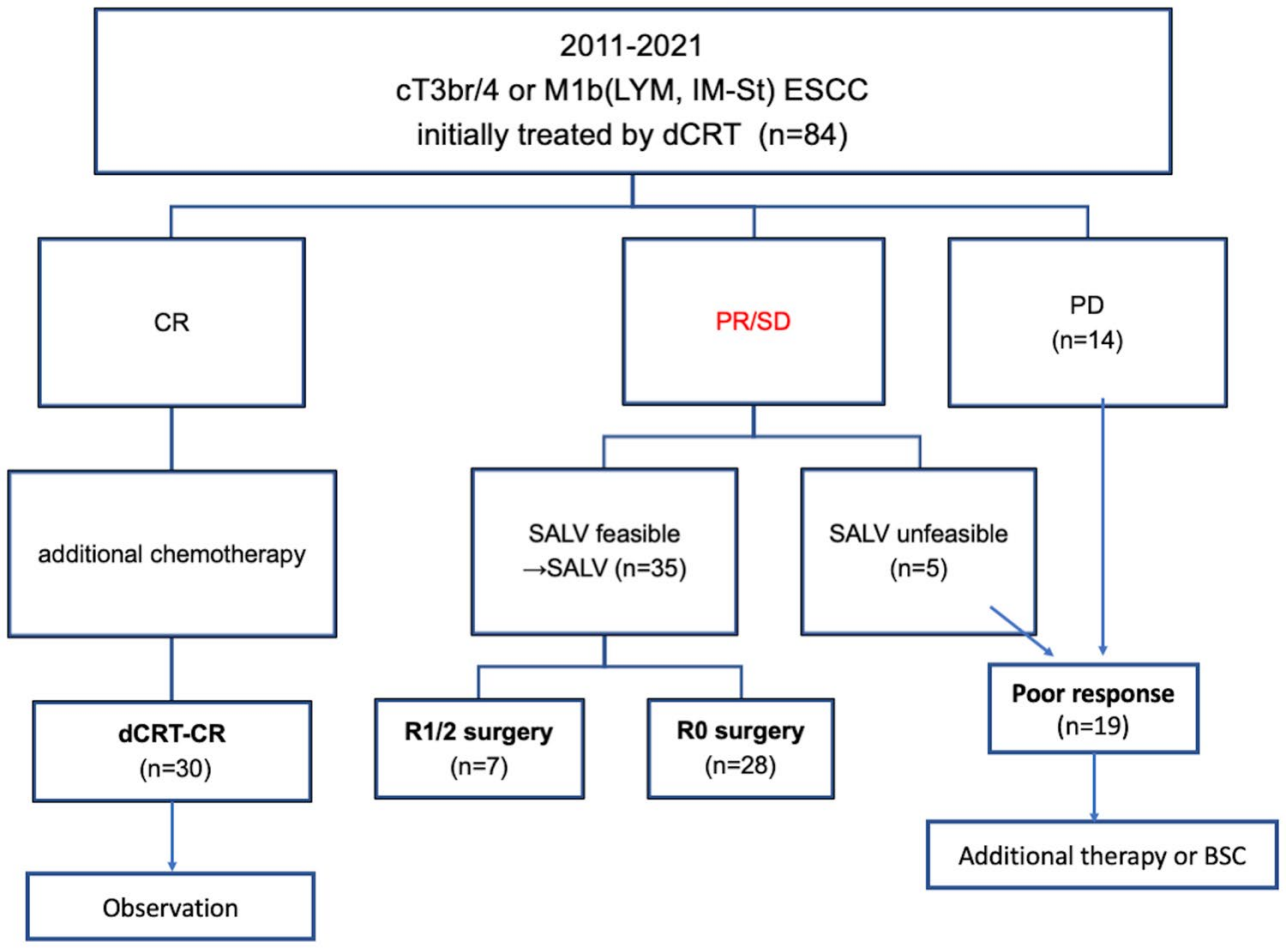
Treatment overview of the patients with cT3br/4 or M1b(LYM, IM-St) esophageal squamous cell carcinoma

Figure 1 shows the treatment overview. Patients diagnosed as having a clinical CR received additional chemotherapy and follow-up evaluations thereafter. For patients diagnosed as having a clinical PR but with suspected or confirmed remnant cancer, surgical resection was generally performed 2–4 months after the completion of dCRT when potentially curative esophagectomy for detected disease was feasible. Patients with stable or progressive diseases were given chemotherapy or palliative therapy.

The basic SALV procedure was a right thoracoabdominal esophagectomy, using either the Ivor Lewis procedure or the McKeown procedure, via right thoracotomy with 1–3 field lymph-node dissection [31]. The Clavien–Dindo scale was used to grade all postoperative morbidities [32].

### Surveillance and definitions of recurrence

Disease recurrence was classified as solely locoregional recurrence (LRR) or distant recurrence. Patients with recurrence at an anastomosis site or a regional lymph node in a cervical (including supraclavicular), mediastinal, or abdominal area were allocated to the LRR group. Patients with recurrence at a distant organ, such as the lung, brain, or liver, were allocated to the distant recurrence group; patients with recurrence in a lymph node surrounding the para-aortic regions were also included in this group. Patients with coexisting locoregional and distant recurrences were assigned to the distant recurrence group.

### Treatment for recurrence

Treatment intents for disease recurrence were divided into surgery, chemo(radio)therapy, radiotherapy, or best supportive care (BSC). We tended to choose resection of metastases when R0 resection was deemed possible. CRT was mainly comprised of 5-FU and CDDP (CF) combined with radiation therapy at a dose of 50–60 Gy. For patients unable to undergo local treatment, such as surgery or CRT/RT, because of extensive recurrence sites, chemotherapy (CTx) comprised mainly of CF and/or taxanes was administered. Patients whose general condition was poor, or who declined treatment for recurrence, received BSC.

### Markers of SIR and nutrition

Data on pre-therapeutic markers of SIR and nutrition were extracted retrospectively from computerized medical records. The SIR and nutritional markers included the neutrophil-to-lymphocyte ratio (NLR), C-reactive protein (CRP)-to-albumin ratio (CAR), lymphocyte-to-CRP ratio (LCR), the systemic immune-inflammatory index (SII), and the prognostic nutritional index (PNI) [21, 22]. The thresholds for NLR, CAR, LCR, and SII were set as the median of values. The cut-off value of the PNI was set as 45. Supplementary Fig. 1 summarizes the method for calculating each marker.

### Statistical analysis

Categorical variables were compared using Fisher’s exact test or the Chi-square test, as appropriate. Continuous variables were compared using Wilcoxon’s rank-sum test or ANOVA, as appropriate. Overall survival (OS) was calculated from the first day of dCRT. Kaplan–Meier survival curves were constructed to estimate survival. We used the log-rank test to estimate the survival difference according to the initial treatment modalities and the response to initial therapies. To identify pre-therapeutic factors which are independently associated with poor OS, we performed a multivariate Cox proportional hazards analysis. Statistical analyses were carried out using JMP 18.0.0 (SAS Institute, Cary, NC).

## Results

### Patient characteristics

Table 2 summarizes the clinicopathological characteristics of the 84 patients with cStage IV ESCC. The median age was 66 years. There were 5 (6.0%) patients with cT3 disease and 79 (94.0%) patients with cT4 disease. The invaded organs were the trachea (*n* = 65), aorta (*n* = 9), and others (*n* = 5). There were 73 (86.9%) patients with cStage IVA disease and 11 (13.1%) with cStage IVB disease. Patients with cStage IVB (*n* = 13) included 6 with pretracheal (106pre) LN metastasis, 5 with paraaorta LN metastasis, and 2 with intramural gastric metastasis.

**Table 2:** Clinical characteristics of the 84 patients with cStage IV esophageal squamous cell carcinoma

Variables	No. of patients (%)
Age, y Median (range)	66 (44–83)
Sex, male	64 (76.2)
Locus Ce/Ut/Mt/Lt	12 (14.3)/20 (23.8)/45 (53.6)/7 (8.3)
cT category, T3/T4	5 (6.0)/79 (94.0)
Extent of invasion, trachea/aorta/others/none	65 (82.3)/9 (11.4)/5 (6.0)/5 (6.0)
cN category, N0/N1/N2-3	15 (17.8)/55 (65.5)/14 (16.7)
cStage, IVA/IVB	73 (86.9)/11 (13.1)
Details of dCRT	
RT dose, 50–50.4 Gy/60 Gy	72 (85.7)/12 (14.3)
Regimen, NDP + 5-FU or TS-1/CF/DCF/Others	58 (69.0)/20 (23.8)/5 (6.0)/1 (1.2)

*ESCC* esophageal squamous cell carcinoma, *Ce* cervical, *Ut/Mt/Lt* upper/middle/lower thoracic, *dCRT* definitive chemoradiotherapy, *NDP* nedaplatin, *CF* *CDDP + 5-FU*, *DCF* docetaxel + CDDP + 5-FU

Figure 1 gives a treatment overview for the entire population. Clinical CR to dCRT was achieved in 30 patients (dCRT-CR group). Forty patients had a partial response to dCRT (PR/SD), and the remaining 14 had progressive disease (PD) after dCRT. Salvage surgery was deemed feasible for 35 of 40 patients with PR/SD disease. Curative (R0) resection was achieved in 28 of the patients who underwent SALV.

Supplementary Table 1 summarizes the characteristics of patients who underwent R0 SALV (*n* = 28). All patients underwent esophagectomy. There was only one surgery-related death (3.6%). There were 15 (53.6%) patients with pT0 (pCR) disease.

### Survival

**Fig. 2:**
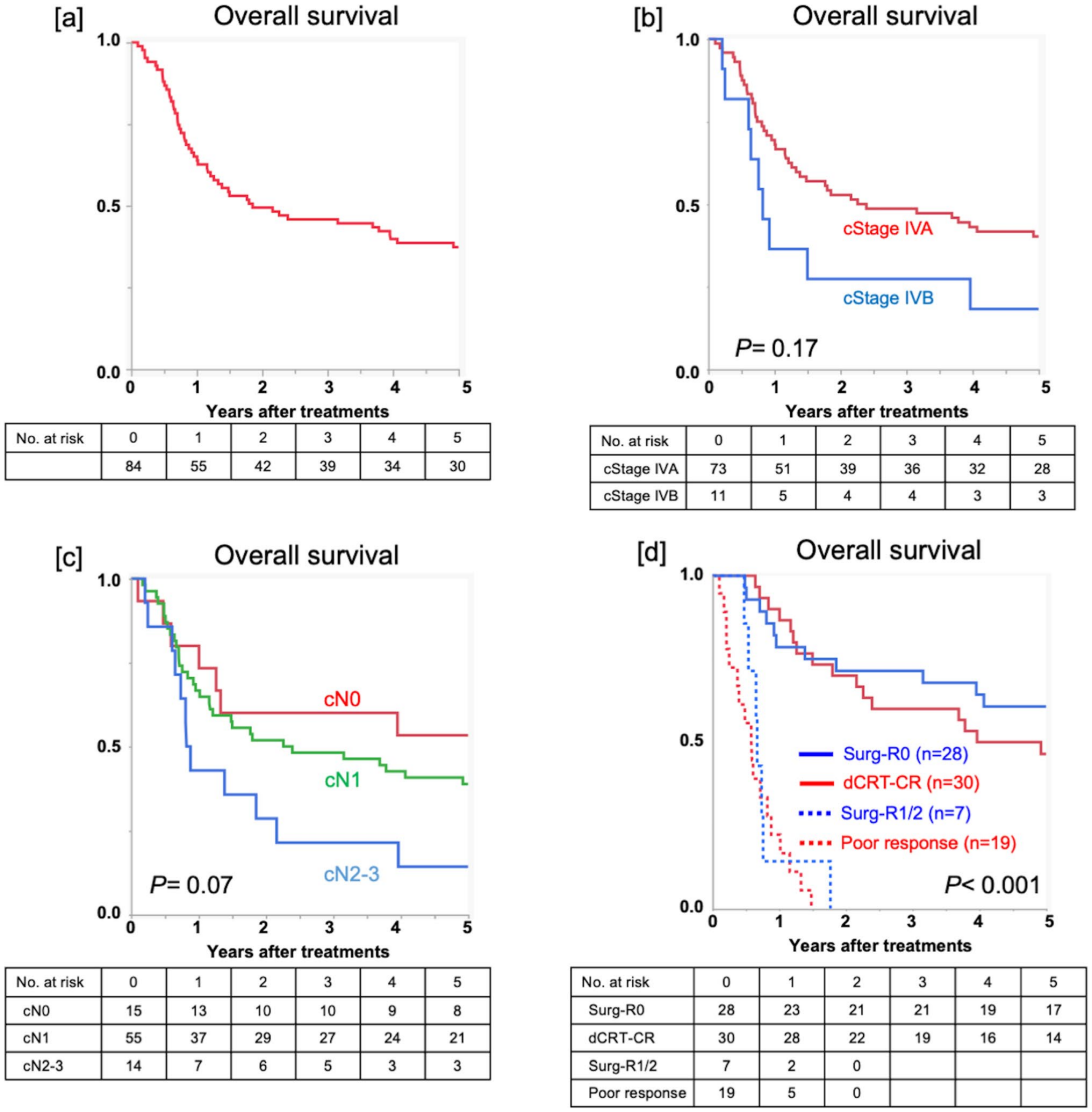
Survival outcomes of the 84 patients. Overall survival curves **a** of the 84 patients, **b** according to cStage (IVA/IVB), **c** according to clinical N stage, and **d** according to the treatment modalities

The 1- and 3-year OS rates of the 84 patients were 65.1% and 45.8%, respectively (Fig. 2a). The 3-year OS of patients with cStage IVA disease was better than that of patients with cStage IVB disease (3-year OS rates; 48.6% vs. 27.3%), but the difference was not significant (*P* = 0.17, Fig. 2b). Moreover, OS curves were not demarcated significantly by cN stage (*P* = 0.07, Fig. 2c).

Patients with clinical CR after dCRT (dCRT-CR group) had a 3-year OS rate of 60.0% (Fig. 2d). Notably, patients who underwent curative surgery after dCRT had almost the same prognosis as patients in the dCRT-CR group (3-year OS rate, 60.7%, Fig. 2d). Patients who underwent non-curative surgery or had a poor clinical response after the initial treatment had extremely poor OS (3-year OS rate, 0%, Fig. 2d).

### Survival according to SIR and nutritional markers

**Fig. 3:**
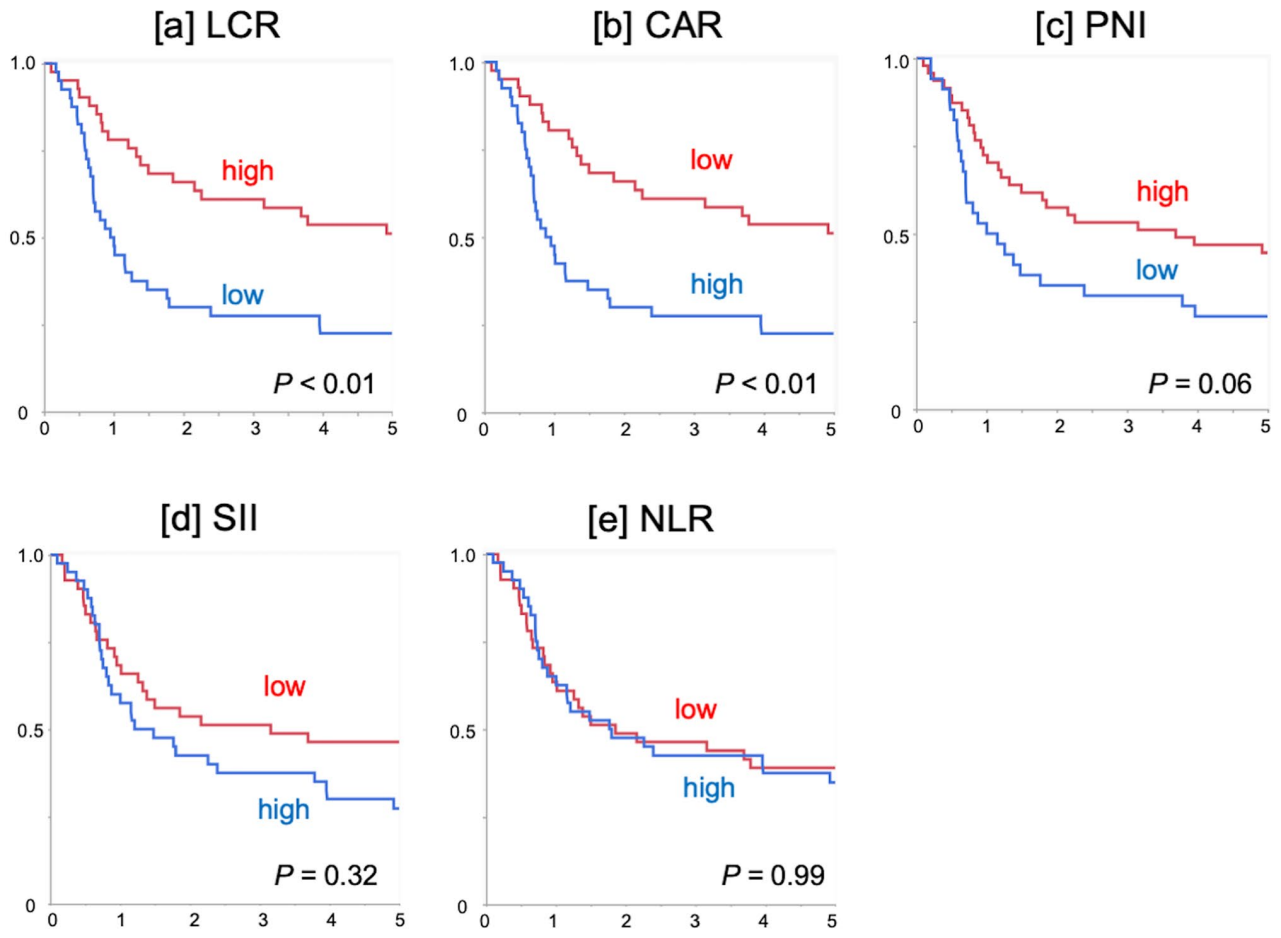
Survival outcomes according to inflammatory/nutritional markers. Overall survival curves were demarcated significantly by **a** the lymphocyte-to-C-reactive protein ratio (LCR; *P* < 0.01) and **b** the C-reactive protein-to-albumin ratio (CAR; *P* < 0.01). Overall survival curves were not stratified significantly by **c** the prognostic nutritional index (PNI; *P* = 0.06), **d** the systemic immune-inflammatory index (SII; *P* = 0.32), or **e** the neutrophil-to-lymphocyte ratio (NLR; *P* = 0.99)

The OS curves were significantly demarcated by CRP-derived markers (LCR and CAR, both *P* < 0.01, Fig. 3a, b). OS curves were also stratified by PNI, but the difference did not reach significance (*P* = 0.06, Fig. 3c). Survival was not well stratified by SII and NLR (Fig. 3d, e). LCR and CAR were significantly associated with the therapeutic response to dCRT (both *P* < 0.01, Table 3), while other markers (PNI, SII and NLR) showed no significant relationship with the response to dCRT (Table 3).

**Table 3:** The response to definitive chemoradiotherapy according to inflammatory/nutritional markers

			dCRT-CR	Poor response	Surg-R0	Surg-R1/2	*P* value (low vs. high)
LCR	Low	*n*	16	4	19	2	0.003
		%	39.0	9.8	46.3	4.9	
	High	*n*	14	15	7	5	
		%	34.1	36.6	17.1	12.2	
CAR	Low	*n*	17	4	19	1	0.001
		%	41.5	9.8	46.3	2.4	
	High	*n*	13	15	7	6	
		%	31.7	36.6	17.1	14.6	
PNI	Low	*n*	11	12	8	4	0.12
		%	31.4	34.3	22.9	11.4	
	High	*n*	19	7	18	3	
		%	40.4	14.9	38.3	6.4	
SII	Low	*n*	14	8	16	3	0.54
		%	34.1	19.5	39	7.3	
	High	*n*	16	11	10	4	
		%	39	26.8	24.4	9.8	
NLR	Low	*n*	14	9	15	3	0.81
		%	34.1	22	36.6	7.3	
	High	*n*	16	10	11	4	
		%	39	24.4	26.8	9.8	

*CR* complete response, *dCRT* definitive chemoradiotherapy, *CAR* CRP-to-albumin ratio, *LCR* lymphocyte-to-CRP ratio, *NLR* neutrophil-to-lymphocyte ratio, *SII* systemic immune-inflammatory index, *PNI* prognostic nutritional index

Univariable analysis and subsequent application of the multivariable Cox proportional hazards model focusing on pre-therapeutic factors revealed low LCR and high CAR to be independently associated with poor OS (Supplementary Table 2). Overall, pre-therapeutic CRP-derived markers were significantly associated with the response to dCRT and the survival of patients who were treated initially with dCRT.

### Outcomes of patients with recurrence after curative treatments

**Fig. 4:**
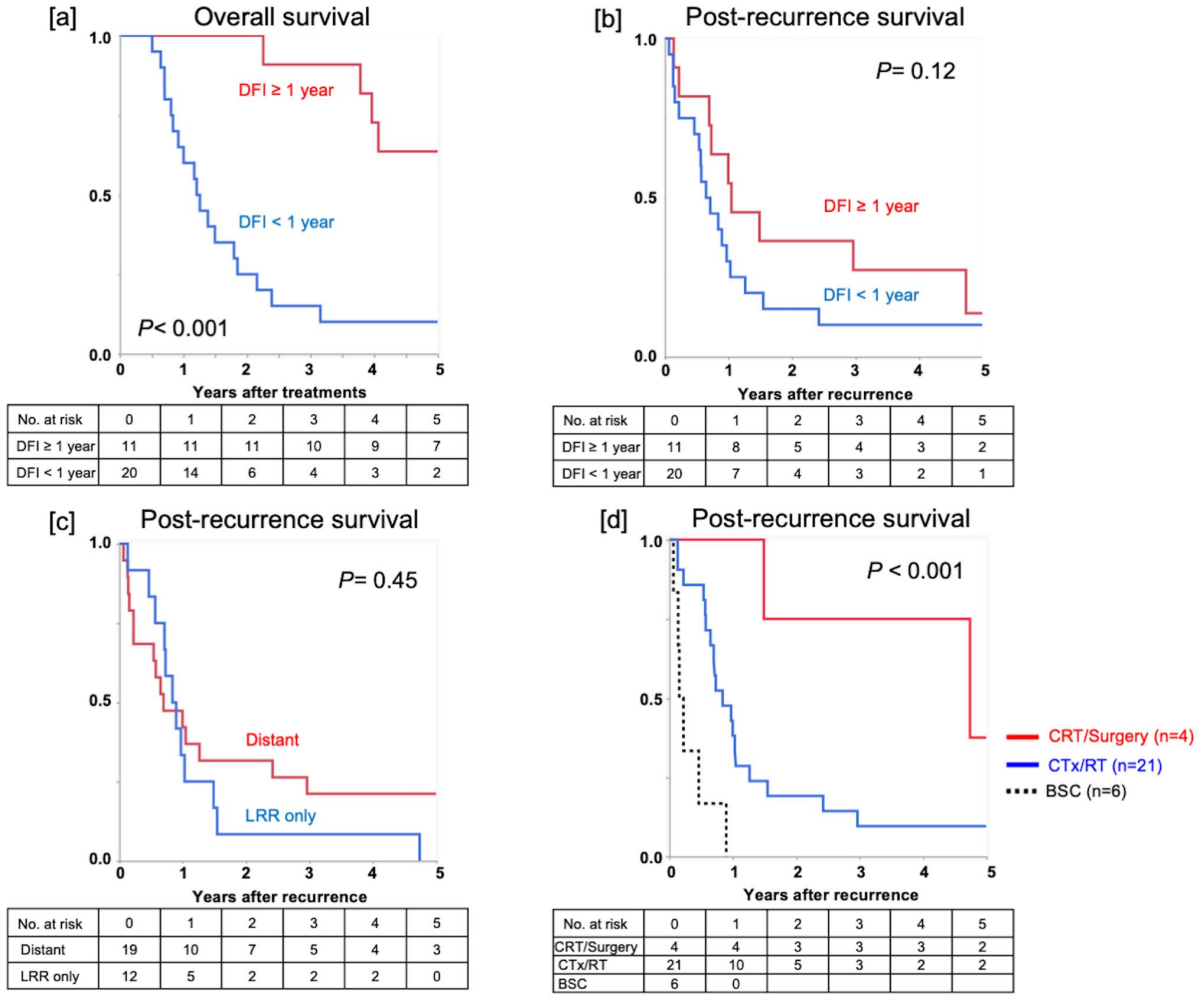
Survival of patients with recurrence. **a** Overall survival was demarcated significantly according to the disease-free interval (≥ 1 year vs. < 1 year). Post-recurrence survival according to **b** disease-free interval, **c** recurrence pattern, and **d** treatment for the recurrence

Finally, we investigated the survival outcomes of patients with recurrence after curative treatments (*n* = 31). Patients with a shorter disease-free interval (DFI) (< 1 year) had significantly lower OS than those with a longer DFI (≥ 1 year) (*P* < 0.001, Fig. 4a). Post-recurrence survival did not differ significantly according to the DFI (*P* = 0.12, Fig. 4b) or recurrence pattern (distant vs. LRR only, *P* = 0.45, Fig. 4c). Treatment for recurrence influenced the PRS significantly (1-year PRS; surgery/CRT vs. CTx/RT vs. BSC, 100.0% vs. 42.9% vs. 0%, *P* < 0.001, Fig. 4d); however, curative-intent treatments were considered only for four patients.

## Discussion

Our study presented the treatment outcomes of dCRT, including SALV, for patients with cStage IV ESCC at our institution, along with an overview of previously published studies. Our findings suggest that dCRT, including SALV, is a feasible treatment option for patients with cStage IV ESCC. Moreover, we demonstrated that SALV provides survival benefits for cStage IV ESCC patients when curative resection is achievable. Notably, CRP-based markers were found to be useful for predicting therapeutic response and long-term outcomes of patients with this tumor entity.

The optimal treatment strategy for patients with locally advanced, unresectable ESCC remains to be established [3, 4]. Since residual or recurrent carcinoma following dCRT is often seen in such patients [10], induction chemotherapy and conversion surgery have recently been employed for this patient population [16, 17]. The JCOG1510 trial is now underway to compare the treatment outcomes of two initial approaches: induction chemotherapy and definitive CRT, for patients with locally advanced, initially unresectable ESCC [33].

The 1- and 3-year OS rates of the 84 patients in this study were 65.1% and 45.8%, respectively. Patients who underwent curative surgery after dCRT exhibited a prognosis similar to that of patients in the dCRT-CR group (3-year OS rate: 60.7%), which is consistent with the findings of another recent study [12]. Previous studies reported that dCRT alone resulted in a 3-year survival rate of 15–33% [6, 9], that NACRT followed by surgery yielded a 3-year survival rate of 25.4% [34], and that induction chemotherapy followed by CRT or conversion surgery achieved a 2-year survival rate of 34.7–55.1% [17], for this patient population (Table 1). Although SALV is known to be highly invasive with considerable mortality, the mortality rate in our study was 3.6% and postoperative complications (≥ IIIa) developed in 39.4% of the patients. These findings suggest that dCRT, including SALV, is a reasonable treatment option for patients with cStage IV ESCC.

These observations also indicate that the survival outcomes of patients with cStage IV ESCC remain poor. Thus, identifying the pre-therapeutic factors associated with the survival outcomes of cStage IV ESCC patients is crucial to improve their prognosis. Response to dCRT is strongly associated with outcome, although response to dCRT cannot be evaluated precisely in the majority of patients. Reliable diagnostic criteria for clinical response remain to be established in clinical practice [35, 36].

Performing surgery in appropriately selected patients is a key factor in improving treatment outcomes. When R0 resection is achieved, these patients are good candidates for salvage esophagectomy following dCRT for initially unresectable, locally advanced T4 esophageal cancer [12, 13]. Although recent studies suggest that endobronchial ultrasonography is useful for assessing resectability of cT4 disease [37], a few modalities can assess resectability accurately, especially in patients with initially cT4 ESCC [2]. Furthermore, pathological CR is thought to be rare in patients who undergo surgery for initially cT4 EC [3, 38]. As such, the relatively aggressive strategy seems reasonable for patients with remnant or recurrent initially T4 ESCC after dCRT [36]. However, previous studies have shown that patients with a good response to preoperative CRT do not experience a survival benefit from subsequent surgical treatment [38–40]. An active surveillance strategy could be a useful therapeutic alternative for ESCC patients who respond well to chemoradiotherapy [41]. Overall, the benefits of SALV for good responders remain controversial.

It is noteworthy that pre-therapeutic CRP-based inflammatory status was associated with both the response to dCRT and survival outcomes in our study. The importance of systemic inflammation and malnutrition in patients with malignancies being recognized increasingly [42]. Previous studies have revealed pre-therapeutic elevated CRP to be significantly associated with the response to CRT for patients who received NACRT followed by surgery for rectal cancer [43, 44]. Furthermore, the CRP-derived systemic inflammation markers LCR and CAR were associated with the response to neoadjuvant therapy in patients with gastrointestinal malignancies [45, 46]. Our findings, together with those of these studies, suggest that CRP plays an important role in predicting the survival outcomes of patients with cStage IV ESCC.

Patients with recurrence after curative treatments generally have poor survival outcomes [47, 48]. Even when R0 resection is performed, recurrence develops in approximately half of all cT4 ESCC patients after surgery [3]. In our study, recurrence developed in about 50% of patients with cCR or after curative resection. We found that a short DFI (< 1 year) and non-curative treatments were associated with poor OS in our cohort. Although curative treatments for recurrence improved PRS, only four patients received curative treatments. Treatment options for recurrence after dCRT remain limited.

Further therapeutic advances are awaited for patients with cStage IV ESCC [49]. Recently, immune checkpoint inhibitors (ICIs) have become a standard treatment for ESCC [50, 51]. Recent clinical trials suggest a promising efficacy and safety profile for the combination therapy of dCRT and ICIs [52, 53]. Clinical trials are now underway to investigate the efficacy and safety of ICIs combined with CRT for ESCC patients [54, 55].

Inter-observer variability has been reported in the clinical diagnosis of the T category of ESCC, particularly for tumors that were deemed unresectable [14, 56]. For example, even T4 ESCCs with tracheobronchial invasion showed variability (tumors that caused only deformity of the tracheobronchial tree or those that extended severely into the tracheobronchial lumen) [57]. In our study, the R0 resection rate was high (28/35, 80%) among patients who underwent SALV, which may be attributed to the relatively small number of patients with tumors that had invaded adjacent organs extensively. While enhanced computed tomography was mandatory for diagnosing T4 disease in our study, endoscopic ultrasonography and bronchoscopy were not required. An appropriate combination of multiple imaging modalities is necessary for accurate T classification, which in turn enables the optimization of treatment strategies for individual patients [17].

The limitations of this study should be considered when interpreting its results. First, while treatment decisions were made during multidisciplinary team meetings, selection bias may have influenced surgical indications and treatment modalities due to the absence of randomization. Second, most of the patients in this study were treated with the NDP and TS-1 regimen for definitive CRT, whereas the CROSS regimen (paclitaxel and carboplatin) has become widely used in Western countries, which may have impacted survival outcomes. Finally, this was a single-institution, retrospective study. We anticipate that a multi-institutional collaborative study with a larger cohort would yield stronger results.

In conclusion, initial dCRT is an appropriate treatment option for patients with cStage IV ESCC. Salvage esophagectomy provides survival benefits for cStage IV ESCC patients only when curative resection is achieved. CRP-based inflammatory markers are useful for predicting therapeutic response and long-term outcomes in this patient population.

## Supplementary Information

Below is the link to the electronic supplementary material.

Supplementary file1 (DOCX 41 KB)

## Abbreviations

ESCC: Esophageal squamous cell carcinoma
dCRT: Definitive chemoradiotherapy
CAR: CRP-to-albumin ratio
LCR: Lymphocyte-to-CRP ratio
OS: Overall survival
CR: Complete response
SALV: Salvage surgery
CS: Conversion surgery
LN: Lymph node
SIR: Systemic inflammatory response
CT: Computed tomography
5-FU: 5-Fluorouracil
NDP: Nedaplatin
PR: Partial response
PD: Progressive disease
SD: Stable disease
LRR: Locoregional recurrence
BSC: Best supportive care
CDDP: Cisplatin
CTx: Chemotherapy
NLR: Neutrophil-to-lymphocyte ratio
SII: Systemic immune-inflammatory index
PNI: Prognostic nutritional index
ICI: Immune checkpoint inhibitor
