# Supplementary material for: Outcomes of patients with clinical stage IV esophageal squamous cell carcinoma treated initially with definitive chemoradiotherapy: a single-institution observational study and literature review
Source: Surg Today. 2025 Jul 4;55(12):1856–67. doi: 10.1007/s00595-025-03087-x (PMC12602661; doi:10.1007/s00595-025-03087-x)
Supplement: Supplementary file 1 — Supplementary file1 (DOCX 41 KB) [file 595_2025_3087_MOESM1_ESM.docx]

| **Supplementary Table 1.**  **Surgical and pathological outcomes of patients undergoing curative salvage surgery (n=28)** | |
| --- | --- |
| Variables | No. of patients (%) |
| Surgical Procedures  Esophagectomy  Lymphadenectomy, 1-2-field/3-field  Morbidity  Anastomotic leakage  Pulmonary (Pneumonia, Empyema)  Chylothorax  Complications*, Grade II/IIIa/IIIb/IV/V  pStage 0-I/II/III/IV/Unknown  Response to dCRT (primary tumor)  Grade 1a/1b/2/3/NA | 28 (100)  21 (75)/ 7 (25)  5 (17.9)  10 (35.7)  2 (7.1)  8 (28.6)/ 8 (28.6)/ 1 (3.6) /1 (3.6)/ 1 (3.6)  13 (46.4)/ 7 (25.0)/ 6 (21.4)/ 1 (3.6)/ 1 (3.6)  2 (7.1)/ 3 (10.7)/ 7 (25.0)/15 (53.6)/1 (3.6) |
| *Clavien-Dindo classification  Abbreviations:  ESCC, esophageal squamous cell carcinoma; dCRT, definitive chemoradiotherapy; NA, not  available | |

| **Supplementary Table 2. Multivariate cox hazards models for overall survival** | | | | | | | | | |
| --- | --- | --- | --- | --- | --- | --- | --- | --- | --- |
| Variables | LCR model | | | CAR model | | | PNI model | | |
|  | HR | 95% CI | *P*  value | HR | 95% CI | *P*  value | HR | 95% CI | *P*  value |
| Age >65  cN category  cN0  cN1  cN2-3  cStage IVB (vs. IVA)  Low LCR  High CAR  Low PNI | 1.21  Ref  1.38  1.88  1.50  1.98 | 0.66-2.11  0.63-2.99  0.74-4.89  0.72-3.17  1.15-3.43 | 0.57  0.39  0.18  0.29  0.02 | 1.19  Ref  1.39  1.89  1.49  1.97 | 0.67-2.12  0.64-3.01  0.73-4.91  0.71-3.15  1.14-3.42 | 0.56  0.40  0.19  0.29  0.02 | 1.40  Ref  1.48  1.91  1.37  1.43 | 0.81-2.45  0.68-3.19  0.74-4.96  0.65-2.88  0.84-2.41 | 0.23  0.32  0.18  0.40  0.18 |
| Abbreviations: LCR, lymphocyte to CRP ratio; CAR, CRP to albumin ratio; PNI, prognostic nutritional index | | | | | | | | | |
